# How to enrich team research in healthcare by considering five theoretical perspectives

**DOI:** 10.3389/fpsyg.2023.1232331

**Published:** 2023-08-10

**Authors:** Julia C. Seelandt, Margarete Boos, Michaela Kolbe, Juliane E. Kämmer

**Affiliations:** ^1^Simulation Center, University Hospital Zürich, Zürich, Switzerland; ^2^Department of Social and Communication Psychology, Institute for Psychology, University of Göttingen, Göttingen, Germany; ^3^ETH Zurich, Zürich, Switzerland; ^4^Department of Emergency Medicine, Inselspital, Bern University Hospital, University of Bern, Bern, Switzerland

**Keywords:** healthcare teams, theoretical perspectives, interaction analysis, group dynamics, small group research

## Abstract

The aim of this paper is to inspire team research to apply diverse and unconventional perspectives to study team dynamics and performance in healthcare settings. To illustrate that using multiple perspectives can yield valuable insights, we examine a segment of a team interaction during a heart-surgery, using five distinct interdisciplinary perspectives known from small group research: the psychodynamic, functional, conflict-power-status, temporal, and social identity perspectives. We briefly describe each theoretical perspective, discuss its application to study healthcare teams, and present possible research questions for the segment at hand using the respective perspective. We also highlight the benefits and challenges associated with employing these diverse approaches and explore how they can be integrated to analyze team processes in health care. Finally, we offer our own insights and opinions on the integration of these approaches, as well as the types of data required to conduct such analyses. We also point to further research avenues and highlight the benefits associated with employing these diverse approaches. Finally, we offer our own insights and opinions on the integration of these approaches, as well as the types of data required to conduct such analyses.

## 1. Introduction

Communication, coordination and leadership in healthcare teams are essential for task performance and patient safety, especially during emergency situations (Tucker and Edmondson, 2003; Manser, 2009; Künzle et al., 2010; Fernandez Castelao et al., 2013; Kolbe and Grande, 2013; Tschan et al., 2014). Teamwork is especially challenging in large hospitals, where turnover rates are high, and for interdisciplinary and interprofessional *ad-hoc* teams lacking the experience of continuously working together as team (Pearce et al., 2006; Nemeth, 2008; St. Pierre et al., 2011; Fortune et al., 2012). Even the willingness and ability to work together do not guarantee success; frequent hurdles are diffuse responsibilities, role conflicts, unsuccessful communication, divergent assumptions about cooperation, skepticism toward other professional groups and the silo mentality that often prevails (Eichbaum, 2018; Rosen et al., 2018; Paige et al., 2019; Kämmer and Ewers, 2022).

How can we foster teamwork in the demanding and ever-changing healthcare environment? While past research has provided valuable insights into the input variables and processes that influence outcomes in healthcare teams (Schmutz and Manser, 2013), we still have much to learn about the temporal dynamics, power dynamics and interprofessional forces at play (Kolbe and Boos, 2019; Anderson et al., 2021). This is partly due to the fact that previous studies have tended to take a particular theoretical perspective when examining healthcare teams: applying what is called the functional perspective, they have examined how selected input factors function to influence group effectiveness (Härgestam et al., 2013; El-Shafy et al., 2018; Schmutz et al., 2018, 2019). However, the theoretical lens we use can influence our findings, and alternative perspectives may be create additional value to studying healthcare teams. Poole and colleagues (Poole et al., 2004) have identified nine interdisciplinary perspectives that can be applied to the study of small groups.

Based on our past research experience, we have noticed that we ourselves tend to act from a silo mentality: We conduct research from primarily one of these perspectives without much considering other perspectives. From our point of view, that “single-mindedness” of sticking to only one theoretical perspective is rather common in healthcare, resulting in reinventing the wheel or disregarding other relevant aspects of teamwork. We believe that using and linking diverse and unconventional perspectives for studying teams in healthcare can broaden our understanding and create additional value. This perspective article does not provide detailed how-to-instruction for conducting team research with each perspective. Instead, our intention is to provide “food for thought” to stimulate team researchers to think out of the box in their next research projects. We therefore present a thought experiment: using segments of the team interaction protocol from a heart-surgery, we demonstrate how we can extract different research questions emerge and offer unique insights when adopting five different perspectives—the functional, conflict-power-status, psychodynamic, temporal, and social identity perspectives. We have selected these five perspectives based on our own research interests, experience, and scientific curiosity; this selection does not claim to be exhaustive. By adopting these perspectives, we aim to shed light on how we can promote effective teamwork (research) in the complex and challenging healthcare environment. We hope that this illustration will offer team researchers who may feel stuck in one viewpoint a fruitful avenue to advance their research, combine certain points of view, and create new research insights that promote teamwork in healthcare. Notably, applying these different perspectives is not limited to healthcare but applicable to teams in other high risk organizations, as has been demonstrated (Hagemann et al., 2011).

## 2. Team interaction during heart surgery

The starting point is the transcript of an audio-recorded team interaction during a scheduled, conventional heart surgery at the University Medical Centre Goettingen (Germany). The surgery was chosen randomly from a control group of 11 surgeries used in another study (Leitsmann et al., 2021; Lehrke et al., 2022).

The surgical team consisted of six members: a primary surgeon (PS, male, age 50), an assisting surgeon (AS, male, 34), a scrub nurse (SN, female, 48), a circulating nurse (CN An, female, 61), an anesthesiologist (An, female, 49), and a perfusionist (HLM, male, 62). The MAGIX Samplitude Music Studio 2017 software (Magix Software GmbH, 2017., Berlin, Germany) was used to record and transcribe the communication, with the transcripts resulting in an Excel 2010 spreadsheet (Microsoft Corporation, 2018). The transcript was segmented into coding units (lines in Table 1) based on syntactic criteria (Kolbe et al., 2016).

**Table 1:** Excerpt of a transcript of an audio-recorded team interaction during a conventional heart surgery with marked perspectives.

Row	Speaker	Transcript of conversation	F	C	P	T	S
16	HLM	Two hundred lie on. %					
17	An	I just get this again forty-six twelve forty-eight. %					
18	PS	Good. #					
19	PS	Can I have the clamp? %					
20	SN	Yes, of course, with pleasure. %					
21	PS	Finally. #					
22	PS	Jesus. %					
23	HLM	Have one always to say it twice? %					
24	PS	Indeed %					
25	CN	It is here underneath. %					
26	PS	Any value to hundred. %					
27	HLM	To hundred. %					
28	An	Forty-eight. %					
29	PS	That’s right. #					
30	PS	This nurse is not qualified for this kind of surgery. #					
31	PS	Jesus. #					
32	PS	As you can plainly see. #					
33	PS	That will never do. %					
34	SN	Well, so I can let myself be replaced by someone else. %					
35	PS	*Susanne, go wash yourself. %					
36	CN	No. #					
37	CN	*Xenia can handle it and stays here. %					
38	An	+Fifty-one to forty-one. #					
39	An	That is two hours that is, that is one hour and %					
40	PS	So, vent is out. %					
41	HLM	Vent is out. #					
42	HLM	Can I suck the /? %					
43	PS	*Xenia does not want to do it anymore. #					
44	PS	She is not able to do that #					
45	PS	She does not feel like it anymore, she said. %					
46	CN	She does %					
47	PS	She does. #					
48	PS	I have heard it, yes %					
49	CN	*Xenia’s back hurts, that’s why. %					

All identified perspectives are marked in the excerpt; the five perspectives are parallel and intertwined. F, functional perspective; C, conflict-power-status perspective; P, psychodynamic perspective; S, social identity perspective; T, temporal perspective. HLM, perfusionist; An, Anesthesiologist; PS, primary surgeon; SN, scrub nurse; CN, circulating nurse; AS, assisting surgeon. #: symbol for separation of two coding units; %: symbol for turn-taking between two speakers; *: pseudonym; /?: not understandable.

The following excerpt (Table 1) captures the beginning of a coronary bypass grafting procedure using conventional extracorporeal circulation. This procedure occurs during a phase of surgery when the aorta is reopened and the patient is under cardiopulmonary support by the heart lung machine. This phase is critical, as the main procedure (bypass grafting) is executed while the patient is in a vulnerable state. At the end of this phase, the heart must pump again without machine support and recover from its protracted metabolic disturbance.

## 3. Five different perspectives for studying team dynamics and team performance

**Figure 1:**
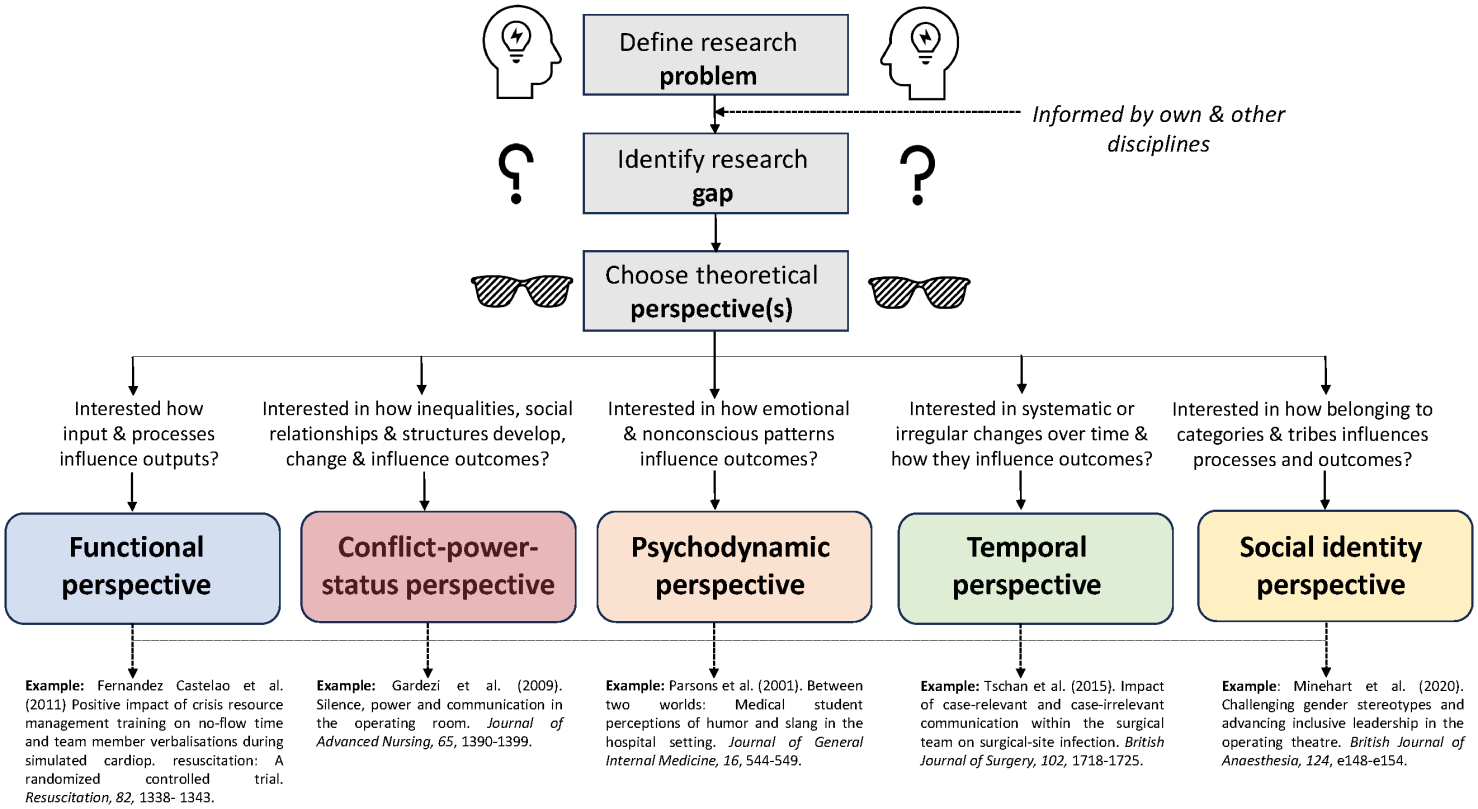
Five perspectives for analyzing team interactions.

In the following sections, we will delve into each of the five different perspectives on studying teams. Per perspective, we will provide a brief overview of its key assumptions, discuss how it could be applied to analyze the excerpt provided and describe for which research goal it is suitable. We also share potential insights and strengths when applying the perspectives to healthcare teams and we outline possible research questions for each perspective in Table 2. All identified perspectives are marked in the excerpt in Table 1. The five perspectives are parallel, intertwined and partly overlapping. Depending on which lens we have on, we can combine up to four different perspectives with each other to analyze this excerpt (Figure 1). For each perspective, different data sources are required (Table 2).

**Table 2:** Descriptions of possible research questions, strengths, and data requirements for each perspective.

Perspective	Exemplary research questions	Strengths for studying healthcare teams/potential insights when applying this perspective to healthcare teams	Potential data sources
Functional perspective	Which processes promote/hinder healthcare team performance in different tasks? How do high performing teams differ from low performing teams in terms of their composition, behaviors and dynamics? Are findings from ad hoc student teams generalizable to organizational real teams?	Ability to predict and explain team performance based on input variables, external conditions and team processes Provide an empirical basis for interventions to improve team performance Dependency of team patterns on task and situational demands Distinction of effective and non effective team routines	Information on input variables, e.g., team size, team composition, stress level, task difficulty, organizational positions, demographics, seniority, expertise Information on processes, e.g., transcripts of interactions Information on the outcome criterion / team performance, e.g., self assessments of satisfaction, quality evaluations, patient survival, information from EHR records, automatically recorded data, document analysis
Conflict-power-status perspective	How does voice and listening behavior differ with respect to role and status in hierarchical teams? How do status hierarchies relate to interprofessional stereotypes? How does tension and microaggression evolve and dissolve within teams? How does psychological safety emerge and change?	Ability to predict subgroup patterns and associated lines of conflict Examination how power is enacted via communication Identification of power relations in interhierarchical and organizationally embedded and/or interprofessional teams	Information on organizational positions, demographics, surface- and deep-level characteristics Information on interpersonal relationships, e.g., trust, cohesion, psychological safety Information in frequency of voice and listening behavior Information on socio-emotional perceptions and reactions
Psychodynamic perspective	How does humor influence communication and performance during surgeries/handover/etc.? Which role plays humor style on team dynamics and perception of teamwork during surgical procedures? How does humor affect teamwork engagement in healthcare teams and team members’ well- being?	Linkage with other perspectives and further differentiations, e.g., feminist perspective, inclusion of hierarchy Possibility to explore different sides of socio-emotional states, e.g., humor with its beneficial and obstructive facets Revelation of general psychodynamic and group dynamic regularities, e.g., conflict escalation, outsiders, scapegoat	Information about surface- and deep-level characteristics, e.g., gender, profession, age Information on processes, e.g., transcripts of interactions Information on socio-emotional perceptions and reactions, and physiological data, e.g., stress, anger
Temporal perspective	How does team interaction evolve over different phases of taskwork (e.g., different phases during surgery)? How do teams adapt from routine to non-routine situations? How do changes in team composition affect team interactions and performance?	Investigation of interaction patterns and (long-term) team development over time Ability to examine team adaptation patterns to task changes and shifts from routine to non-routine situations Provide an empirical basis for team interventions to improve team development	Information on team characteristics, e.g., developmental stage, task type Information on processes, e.g., transcripts of interactions (e.g., time-stamped data) Information on physiological data, e.g., stress, anger, and performance measures
Social identity theory	How does teamwork with ingroup members differs from teamwork with outgroup members? How do team faultlines and stereotypes affect quality of care? How to promote teamwork between subgroups?	Provide insights into effects of self- and other-categorization and stereotypes Ability to predict subgroup patterns Provide an empirical basis for team interventions to improve team identification	Information about surface- and deep-level characteristics, e.g., gender, profession, age Information on processes, e.g., transcripts of interactions Information on socio-emotional perceptions and reactions

EHR, Electronic Health Record.

### 3.1. The functional perspective

Scholars taking a functional perspective assume that groups are goal-oriented and that inputs (e.g., the group task) and/or processes (e.g., communication) influence group performance (e.g., productivity, effectiveness, satisfaction) as well as external factors (e.g., organizational structures, regulations), all of which can be evaluated (Poole et al., 2004; Hollingshead et al., 2005). Their research goal is to identify relevant group features and behaviors (such as certain communication or coordination patterns) that promote or hinder group performance (Fernandez Castelao et al., 2011; Kolbe et al., 2014; Willmes et al., 2022). For example, one result obtained by taking this perspective is that closed-loop-communication (CLC), where a command is followed by a checkback and closing the loop (Härgestam et al., 2013; El-Shafy et al., 2018), correlates with higher task performance (e.g., lower hands-off time in resuscitation, better adherence to guidelines) and thus higher patient safety (Salas et al., 2008). Research from a functional perspective is suited to inform the testing of certain interventions (e.g., checklists)(Lingard et al., 2008; Russ et al., 2015), the development of interventions to improve team performance, such as crisis resource management principles (Oberfrank et al., 2019), different mnemonics to help teams quickly organize themselves (e.g., 10 s for 10 min) (Rall et al., 2008) and briefing and debriefing interventions (Lingard et al., 2008, 2011; Russ et al., 2015).

Consider the episode in lines 19–21, where the surgeon asked the scrub nurse to get the clamp and the nurse acknowledges it. Instruction-reaction episodes such as this one may be analyzed in terms of their completeness by comparing them to the “ideal” CLC cycle (Tschan, 1995, 2002). Additionally, one could check which internal and external factors prevent the correct implementation of the CLC cycles. This analysis could reveal the proportion of standard vs. non-standard forms of CLC and relate it to outcome measures such as the number of followed instructions or patient survival (Marzuki et al., 2019).

Another functional approach to the excerpt would be to code the content of utterances with an established coding scheme (i.e., assign pre-defined behavior and communication codes to sequences of the interaction). For example, researchers may code case-relevant communication (CRC) such as ‘instructions’, versus case-irrelevant communication (CIC) such as chitchatting (Seelandt et al., 2014; Lehrke et al., 2022). The proportions and patterns of CRC to CIC episodes could then be set in relation to outcome variables such as satisfaction with teamwork or team effectiveness [e.g., surgical site infections (Widmer et al., 2018)].

In sum, researchers interested in crucial inputs and processes influencing team performance outcomes are advised to adopt this perspective. Exemplary research questions as well as recommendations for data sources are highlighted in Table 2. However, focus on the functional perspective is often limited to “team performance,” disregarding other important outcomes such as well-being or system maintenance.

### 3.2. The conflict-power-status perspective

Scholars taking a conflict-power-status perspective assume that resources, status, and power are unequally distributed within groups (Poole et al., 2004; Sell et al., 2004). Their research goal is to understand how these inequalities, social relationships and associated group structures develop and change, and how they influence group processes (e.g., conflict management) and outcomes [e.g., member satisfaction (Poole et al., 2004)] Healthcare teams seem to be a logical place for adopting the conflict-power-status perspective (Janss et al., 2012) given that differences in (legitimate) power and occupational status are paramount (Hollenbeck et al., 2012). In our operating room team example, surgeons, anesthesiologists, perfusionists and nurses may each have certain explicit positional status and power, yet also have implicit, subtle and relational status and power based on experience, tenure and relationships (Yule et al., 2006; Gardezi et al., 2009).

Consider the episode in lines 30–37, where we witness how the surgeon expressed his dissatisfaction with the nurse, whereupon the nurse offered to be substituted by another nurse. While the surgeon agreed with this suggestion, instructing another nurse to enter, the circulating nurse overtly objected, instructing the first nurse to stay. Applying the conflict-power-status perspective to analyzing the excerpt offers the possibility to study how power is explicitly and implicitly enacted (e.g., by examining who instructs whom), how open (vs. subtle) conflicts are enacted, or which coalitions exist. Assumptions and discussion about responsibilities, performance or authority are a frequent source of tension in the operating room (Lingard et al., 2002, 2004). Tension, frustration and conflict influence the quality of team interactions. For example, while observing disrespectful behavior may cause team members to speak up with a concern, a general lack of psychological safety or of inclusive language may impede live-saving speaking up (Edmondson, 2003; Raemer et al., 2016; Weiss et al., 2018; Krenz et al., 2020; Vauk et al., 2022).

As healthcare is more and more provided by multidisciplinary teams whose professional members each have a unique identity with potentially differing priorities, roles and expectations of how care should be delivered, micropolitical interests have to be negotiated (Taplin et al., 2015; Kolbe et al., 2019). Politics refers to the use of power, authority and influence and is a relational process between people and within teams (Rogers et al., 2020). This excerpt also gives rise to the possibility to assess emotional reactions to and satisfaction with the manner in which hierarchy is acted out and which role sarcasm, humor and irony play in such power games (Krenz et al., 2019; Long et al., 2020; Koopman et al., 2023; Weiss et al., 2023).

In sum, researchers interested in understanding status and power inequalities, group structures and their impact on team performance outcomes are directed to this approach. However, research strongly following the conflict-power-status perspective may require high levels of reflexivity from researchers who have their own personal views on conflict, power and status dynamics.

### 3.3. The psychodynamic perspective

Scholars taking a psychodynamic perspective assume that emotional and nonconscious processes exist within all human groups which impact their interactions and task performance (Mcleod and Kettner-Polley, 2004). Their research goal is to understand emotions and unconscious patterns of behavior (Mcleod and Kettner-Polley, 2004). To increase team performance, these nonconscious processes have to be brought to team members’ conscious awareness (Mcleod and Kettner-Polley, 2004). One of these nonconscious processes is humor (Newirth, 2006).

Humor can have different functions. On the one hand, humor takes on a conducive role and positive humor has many benefits. It may alleviate tension, fatigue, and improve work relationships (Crowe et al., 2016). Humor also has a relaxing function and can buffer the negative effects of stress on health and well-being (Martin, 1996; Karl et al., 2007). In addition, humor reduces perceived stress and the likelihood of burnout and strengthens resilience (Murden et al., 2018; Rose et al., 2021). On the other hand, humor and jokes can serve as a gateway for prejudices or to devalue other individuals (Prusaczyk and Hodson, 2020). Humor can be employed to define the status quo of a group or to maintain and consolidate the hierarchy within a team (Hodson and Prusaczyk, 2021). Interestingly, gender often plays a role regarding the negative form of humor, with women being the target of sexualized humor (Tabassum and Karakowsky, 2022).

Consider the episode in lines 33–49, where two female nurses and a male surgeon were part of what appears to be a humorous interaction. The surgeon questioned the performance of one nurse and made it sound as if she could not do her job and did not feel like doing it. He used a very colloquial formulation (“She does not feel like it anymore”) and this humorous interaction contains an ambiguity (which is typical for humor). He may have used humor to “soften” his message and to offer a more or less suitable excuse for what could otherwise be perceived as rude (Ringblom, 2022). Or, he may have used humor to put women (the nurses) in an inferior position and to maintain a gender- and/or status-based ingroup-outgroup distinction (Ringblom, 2022).

In this episode, it would be also interesting to examine the speaking up behavior of the participants. One might explore to what extent negative humor influences the speaking up behavior of the ironized group (the nurses) or the whole group and to clarify whether this behavior could be a hindrance or even beneficial for further speaking up (Parsons et al., 2001; Vauk et al., 2022).

Numerous studies on emotions, stress management, and burnout among health-care workers exist (e.g., during COVID-19 emergency (Di Giuseppe et al., 2021)) with only few studies on humor and well-being (e.g., effect of humor on nursing professionals’ well-being (Navarro-Carrillo et al., 2020)), albeit unrelated to healthcare teams. Therefore, investigating the role of humor in healthcare teams and its relation to well-being and speaking-up could not only be promising but applying the psychodynamic perspective may provide desired guidance for researchers who wish to identify emotional and nonconscious processes within teams and their impact on further interactions and performance. However, team research mainly following the psychodynamic perspective may struggle with the multiple and even conflicting socio-emotional processes, e.g., humor may have both a beneficial and obstructive impact (Tschan et al., 2015).

### 3.4. The temporal perspective

Scholars taking a temporal perspective assume that groups are systems that evolve over time and in which change is generic and arises across multiple time scales (Arrow et al., 2004). Their research goal is to discriminate changes that are systematic or even regular from changes that are episodic and particular. They also aim to understand how groups systematically change over time and how this group development can be described, explained and modeled (Harvey et al., 2023). On the micro level, the patterning of interaction in groups comes into focus and how these dynamics relate to relevant other factors like group performance, team member satisfaction etc.

Healthcare teams exhibit dynamics on both levels, the meso level of the dynamics of the team as a whole as well as the micro level of interaction patterns. For example, guideline-oriented teamwork as it is prevalent in resuscitation teams entrains the dynamics of the group as a whole, measurable by the degree of guideline adherence (Fernandez Castelao et al., 2015). Another example is an interaction pattern on the micro level who assumed that groups shift from behaviors focused primarily on the task to behaviors relating to the socio-emotional requirements of the group (Bales, 1950). This can be explained by Bales’ equilibrium model (Bales et al., 1953), which claims that a group must keep a balance between task-oriented and socio-emotional needs, in order to be successful. However, socio-emotional behavior might merge into CIC which, at some point, might cause distractions for team members and impair surgical outcomes (Tschan et al., 2015; Wheelock et al., 2015). Other temporal patterns found in healthcare teams are adaptation processes where implicit vs. explicit coordination mechanisms are situationally adapted to routine vs. non-routine requirements of the task (Burtscher et al., 2011; Riethmüller et al., 2012).

Consider the episode in lines 19 to 45, where we can apply the basic distinction between CRC and CIR outlined previously in the functional perspective. From the temporal perspective, we can state that this episode is composed of different micro episodes swaying from CRC and CIC communication. This shift back and forth between CRC and CIC creates a non-random interaction pattern relating systematically to task performance and well-being functions of the team. It would also be interesting to explore whether the CIC utterances in this group serve the tension-reduction function assumed in the equilibrium model (Bales, 1950) or – on the contrary – induce interpersonal conflict and thus impair team performance. Thus, researchers aiming at detecting and describing dynamic patterns in teams and relating these patterns to diverse functions of a team are recommended to apply the temporal perspective. However, research mainly driven by the temporal perspective may involve risks that such too fine-grained analyses of micro processes leaving out structural conditions on the meso (team as a whole) and macro (embedding organization, socio-political system) levels.

### 3.5. The social identity perspective

Scholars taking the social identity perspective assume that relations between large-scale social categories as nations, cultural groups etc. exist and analyze the cognitive aspects of self- and other-classifications of social groups and group membership (Hogg et al., 2004). Social identity is “the individual’s knowledge that he belongs to certain social groups together with some emotional and value significance to him of this group membership” (Tajfel, 1972, p. 292). These scholars’ research goal is to describe how the categorization of self and others define group memberships, the construction of group norms and the enactment of these norms in group and intergroup behavior (Turner, 1985; Turner et al., 1987; Hogg et al., 2004). For example, belonging to different professional groups impacts how healthcare team members react to inclusive language and speak up (Weller et al., 2014; Weiss et al., 2018). Even more, gender stereotypes woefully impact team interaction in the OR (Pattni et al., 2017; Minehart et al., 2020). That means, in a given situation a specific social category – in our example physician or nurse – might be salient due to the context, here the heart surgery in the operating theatre.

Consider the episode in lines 30–37, one could describe the interaction between the surgeon and the nurse(s) as an intergroup situation, primarily on the interprofessional dimension physician versus nurse. In the surgeon’s utterance “this nurse …,” the (scrub) nurse Xenia is addressed as a member of her social category. The physician addresses her not as an individual, but through the lens of the stereotype “nurse” which means the person become depersonalized. Although the nurse is present in the situation and working at the operating desk with the surgeon, she is addressed in the third person, not with her name but with her professional classification. Her colleague, the circulating nurse, immediately comes to her defense, says her name (“Xenia”) and provides cover. One could even go thus far that the circulating nurse tries to annulate the relational communication on the collective level (differentiating “we” from “them”) by trying to get back to the interpersonal level of “Xenia” interacting with the other team members. Applying the social identity perspective to analyze the excerpt, we could identify which social categories are salient in this team. Besides the interprofessional categorization – physician vs. nurse – there is also the gender-dimension, man versus women. In the ironic, sarcastic or even aggressive way the surgeon comments on the competencies of the nurse, one could even see a categorization on the dimension of hierarchy which parallels the other two dimensions. Thus, the social identity perspective provides theoretical guidance if research questions focus on the conditions and effects of identification with the team, with subteams or the discrimination or even competition toward other teams or larger social units and categories. However, research mainly based on the social identity perspective risks overlooking the variety and creativity of the behavior of team members as individual persons (rather than as members of social categories).

## 4. Discussion

How team members work with one another, with other teams, with patients and their relatives impacts everybody’s well-being (Pronovost, 2013; WHO, 2021). Teams are not black boxes and exploring how team members manage teamwork in the complexity (Lingard et al., 2004) of healthcare systems will help identifying how to support them best (Kolbe and Boos, 2019; Anderson et al., 2021). Team science provides orientation, theoretical and methodological guidance, and resources for how to study teamwork. Reflecting on how we use these methodologies is important for drawing conclusions. In our perspective article we attempted to illustrate how our theoretical lens influences how we study teamwork in healthcare. It seems fascinating that a brief sequence of an operating room team conversation can be explored from many perspectives with varying foci: performance, power, identity, time and many more. Our purpose was to highlight the benefits of leaving static research behind but use the existing versatility of team theory to inspire team research in healthcare and other high responsibility domains.

Whether conscious or unconscious, our choice of a particular theoretical lens both sharpens our focus and leaves us blind to possible other phenomena. Applying the problem-gap-hook heuristic (Lingard and Watling, 2021), we hope that our illustration will provide guidance for studying teams in healthcare in identifying the problem, gap, and hook.

### 4.1. Identifying the problem

What is the problem that matters? Exposure to disrespectful team members? Impeded patient safety when team members do not share or listen to safety concerns? Lack of clarity on whether or how heart team meetings work? Precisely identifying the problem at hand is important because it will guide which theoretical lens(es) may fit best for studying it. For example, if in our heart surgery example (Table 1) the perceived stress and reduced well-being of the operating room team were problematic, applying not only the functional but also the conflict/power/status and psychodynamic perspectives might be fruitful and direct researchers to studying the tensions, potential toxic functions of humor and other forms of disrespectful communication in the OR (Lingard et al., 2004). Notably, the problem is not the same as the research gap.

### 4.2. Identifying the gap

What is already known about the problem and what is the current gap in the research, precisely? From our experience, a research review beyond the scope of one discipline and one theoretical perspective typically reveals plenty of existing research that will help sharpen the research question and methods. For example, when studying voice in healthcare teams, reviewing the voice literature in organizational behavior and psychology yields a variety of concepts, methods and results applicable to healthcare teams (Heaphy et al., 2022; Li and Tangirala, 2022). For broadening the research beyond healthcare, the dimensional model of Hagemann allows for identifying similarities, differences, and application (Hagemann et al., 2011). Notably, identifying the research gap can be a challenging step as research from different theoretical lenses and disciplines is frequently published in different kinds of journals; researchers may benefit from leaving the comfort zones of their field’s journals.

### 4.3. Identifying the hook

Why does the research gap and the chosen approach to closing it matter? The team research perspectives described in this article can be a considerable hook (Figure 1): A problem may be studied from a *different perspective*. For example, while voice in healthcare teams has typically been studied from the conflict/status/power perspective, applying a psychodynamic perspective may discover unconscious voice/silence patterns (Foulk et al., 2016). Alternatively, a problem may be studied *combining different theoretical perspectives*. For example, knowledge on facilitating voice in healthcare teams may be enhanced by combining the conflict/status/power with the psychodynamic perspective, linking power, status, patterns and voice communication (Weiss et al., 2017, 2018, 2023; Lemke et al., 2021). As another example, a behavioral observation study on teamwork and communication within surgical teams has shown that more case-irrelevant communication including humor during wound closure is related to worse patient outcomes, whereas case-relevant communication during the whole surgery seems to be a protective factor against surgical site infections (Tschan et al., 2015). This impressive study evolved through combining the psychodynamic, the temporal, and the functional perspectives. Further combinations of theoretical perspectives are conceivable: combining the functional with the conflict-power-status perspective may enrich our understanding of crucial relational aspects improving or undermining team effectiveness (Janss et al., 2012; Weiss et al., 2023). Combining the functional with the temporal perspective (Fernandez Castelao et al., 2015) to find out how effective and less effective behavioral patterns emerge and can be supported or avoided may be fruitful, e.g., by training or intervention. Similarly, the social identity perspective may fit well to the conflict-power-status and functional perspectives for exploring the effects of stereotyping on team and leadership effectiveness as well as on patient safety (Weller et al., 2014; Pattni et al., 2017; Minehart et al., 2020).

## 5. Conclusion

Thus, reflecting on which theoretical lenses we apply when studying dynamics in healthcare sharpens our focus. It sharpens what we are looking at, how we are looking at it and what literatures and methodologies we will use to inform our research (Weingart, 1997; Edmondson and Mcmanus, 2007).

Our analysis has limitations. First, there are more theoretical perspectives to studying team dynamics than we have discussed here (Poole et al., 2004). Our discussion is a starting point rather than a comprehensive exploration of each perspective. Further research is required; in particularly with respect to equity, diversity and inclusion in healthcare teams (Rosenkranz et al., 2021). For example, combining the psychodynamic with the so-called feminist perspective might yield important insights into how gender and privilege are enacted in team interaction (Minehart et al., 2020; Tramèr et al., 2020; Hochstrasser et al., 2022; Zwicky et al., 2022).

Second, we did not discuss why some theoretical perspectives (e.g., functional perspective) may, explicitly or implicitly, have been used more often than others (e.g., temporal perspective). Methodical constraints and required effort in accessing temporal data may play a significant role and new advances in collecting temporal team interaction may help (Weiss et al., 2023). Third, particularly the science of healthcare teams has to factor in two seemingly distinct mindsets of what constitutes “good data”: On the one hand, psychological and team science involve expertise in recording and describing social phenomena, such as perceptions, attitudes, or behavior in teams (Weingart, 1997; Brauner et al., 2018). Valid measurement instruments are developed to measure these data precisely and to be able to use them in behavioral observations, surveys/questionnaires, and interviews. This type of data collection may at first seem unusual to medical researchers, who, on the other hand, rely on more “objective” data such as physiological values. On the other hand, medical science considers randomized clinical trials the state of the art (Benson and Hartz, 2000). They may represent a particular form of the functional perspective and explain why much research on healthcare teams does indeed apply a functional perspective. In our view, it is precisely the diversity of interdisciplinary methods that would allow for other, new angles for research. Studying healthcare teams by translating and applying methods from medicine and nursing, organizational behavior, psychology, mechanical engineering and informatics seems now easier than a decade ago and allows for new avenues and methodologies for studying healthcare team dynamics (Rosen et al., 2014, 2018; Hałgas et al., 2023; Weiss et al., 2023). While we are aware of the enormous effort involved in planning, conducting and analyzing healthcare team research with any of the discussed perspectives, we believe in their potential for improving teamwork and patient care.

## Data availability statement

The original contributions presented in the study are included in the article/supplementary material, further inquiries can be directed to the corresponding author.

## Ethics statement

Ethical review and approval was not required for the study on human participants in accordance with the local legislation and institutional requirements. Written informed consent from the patients/participants or patients/participants legal guardian/next of kin was not required to participate in this study in accordance with the national legislation and the institutional requirements.

## Author contributions

All authors listed have made a substantial, direct, and intellectual contribution to the work and approved it for publication.

## Funding

Open access funding was provided by ETH Zurich.

## Conflict of interest

The authors declare that the research was conducted in the absence of any commercial or financial relationships that could be construed as a potential conflict of interest.

## Publisher’s note

All claims expressed in this article are solely those of the authors and do not necessarily represent those of their affiliated organizations, or those of the publisher, the editors and the reviewers. Any product that may be evaluated in this article, or claim that may be made by its manufacturer, is not guaranteed or endorsed by the publisher.
